# Prion Protein (PrP) Knock-Out Mice Show Altered Iron Metabolism: A Functional Role for PrP in Iron Uptake and Transport

**DOI:** 10.1371/journal.pone.0006115

**Published:** 2009-07-01

**Authors:** Ajay Singh, Qingzhong Kong, Xiu Luo, Robert B. Petersen, Howard Meyerson, Neena Singh

**Affiliations:** Department of Pathology, Case Western Reserve University, Cleveland, Ohio, United States of America; Massachusetts General Hospital and Harvard Medical School, United States of America

## Abstract

Despite overwhelming evidence implicating the prion protein (PrP) in prion disease pathogenesis, the normal function of this cell surface glycoprotein remains unclear. In previous reports we demonstrated that PrP mediates cellular iron uptake and transport, and aggregation of PrP to the disease causing PrP-scrapie (PrP^Sc^) form results in imbalance of iron homeostasis in prion disease affected human and animal brains. Here, we show that selective deletion of PrP in transgenic mice (PrP^KO^) alters systemic iron homeostasis as reflected in hematological parameters and levels of total iron and iron regulatory proteins in the plasma, liver, spleen, and brain of PrP^KO^ mice relative to matched wild type controls. Introduction of radiolabeled iron (^59^FeCl_3_) to Wt and PrP^KO^ mice by gastric gavage reveals inefficient transport of ^59^Fe from the duodenum to the blood stream, an early abortive spike of erythropoiesis in the long bones and spleen, and eventual decreased ^59^Fe content in red blood cells and all major organs of PrP^KO^ mice relative to Wt controls. The iron deficient phenotype of PrP^KO^ mice is reversed by expressing Wt PrP in the PrP^KO^ background, demonstrating a functional role for PrP in iron uptake and transport. Since iron is required for essential metabolic processes and is also potentially toxic if mismanaged, these results suggest that loss of normal function of PrP due to aggregation to the PrP^Sc^ form induces imbalance of brain iron homeostasis, resulting in disease associated neurotoxicity.

## Introduction

Prion protein (PrP) is a ubiquitously expressed cell surface glycoprotein linked to the plasma membrane by a glycosylphosphatidylinositol (GPI) anchor. Although several lines of evidence support the obligate role of PrP in animal and human prion disorders, relatively less is known about the normal function of this protein [1], [2]. Attempts to understand the physiological function of PrP by generating PrP knock-out (PrP^KO^) transgenic mouse lines have been futile since the mice do not develop an overt phenotype other than resistance to prion disease [3]. The limited information on possible physiological function(s) of PrP has been obtained from the Zurich 1 and Edinburgh PrP^KO^ mice since these do not up-regulate Doppel, a PrP homologue that induces cerebellar degeneration [4], [5]. Most of the investigations on these and other mouse models have focused on brain function since PrP is most abundant on neuronal cells and is therefore likely to alter brain function by its absence. A similar loss of function due to aggregation of PrP to the disease causing PrP-scrapie (PrP^Sc^) form is also likely to alter neuronal function, partially explaining the pathogenesis of prion disorders and justifying the focus [2]. Several important facts have emerged from these studies; PrP^KO^ mice show altered circadian rhythm and sleep pattern, increased susceptibility to neuronal damage by oxidative stress and cerebral ischemia, neurotoxicity by expression of Doppel and N-terminally truncated PrP, increased predilection to seizures, motor and cognitive abnormalities, reduced synaptic inhibition and long term potentiation in the hippocampus, altered development of the granule cell layer, mis-regulation of the cerebellar network, and age-dependent spongiform change with reactive astrogliosis [5]–[10]. Examination of peripheral organ systems reveals impaired ability of hematopoietic progenitor cells of PrP^KO^ mice to colonize when transplanted to irradiated recipient mice [11], and poor recovery of PrP lacking animals from experimentally induced anemia [12]. The multiplicity of observations attributed to the absence of PrP in the same transgenic mouse line suggests its involvement in an essential function with broad implications. Though attractive, this hypothesis has remained untested.

Recently, we demonstrated that PrP mediates iron uptake and transport in human neuroblastoma cells [13], and aggregation of PrP to the disease causing PrP-scrapie (PrP^Sc^) form induces an imbalance of iron homeostasis in prion disease affected human, hamster and mouse brains [14]. Here, we extend the cell based studies to mouse models to understand the role of PrP in iron metabolism *in vivo*. Using the Zurich 1 PrP^KO^ and matched wild type (Wt) mice expressing normal levels of PrP as experimental models, we demonstrate that PrP^KO^ mice show a phenotype of systemic iron deficiency and altered iron homeostasis compared to Wt controls. The underlying cause of this abnormality is impaired transport of iron from the duodenal epithelium to the blood stream, and a similar defect in iron uptake by parenchymal cells of various organs and hematopoietic precursor cells. The iron deficient phenotype of PrP^KO^ mice is rescued by re-introducing PrP on the PrP^KO^ background, indicating a functional role for PrP in iron uptake and transport. Since iron is an essential element that is necessary for survival but can be extremely toxic if mis-managed [15], these results have significant implications for understanding the physiological function of PrP in cellular iron metabolism, and the pathological implications thereof due to its aggregation to the PrP^Sc^ form, the principal agent responsible for prion disease associated neuronal death.

## Results

### Major organs of PrP^KO^ mice show a phenotype of relative iron deficiency

**Figure 1:**
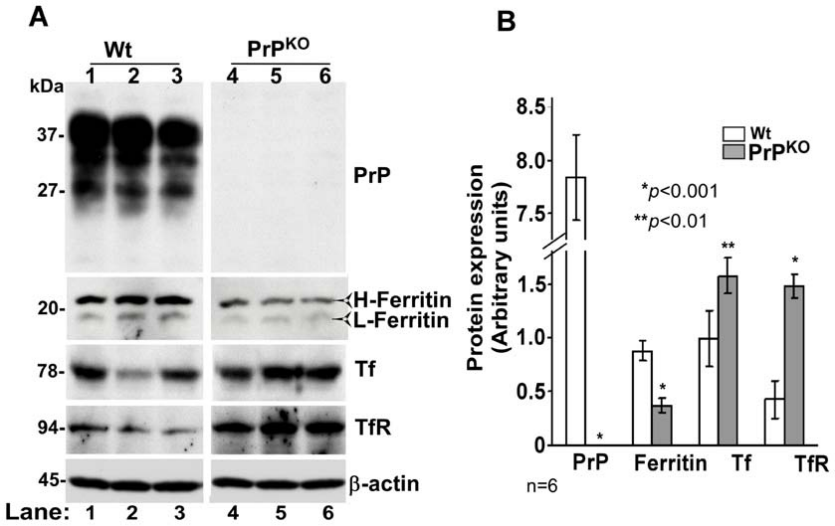
Brain homogenates of PrP^KO^ mice show evidence of iron deficiency. (A) Immunoblot analysis of Wt and PrP^KO^ brain homogenates with PrP specific antibody 8H4 shows the expected glycoforms of PrP in Wt samples migrating between 27–37 kDa, and no reaction with PrP^KO^ samples as expected (lanes 1–6). Immunoreaction for ferritin, Tf, and TfR shows significantly less ferritin, and more Tf and TfR levels in PrP^KO^ samples compared to Wt controls (lanes 1–6). Reaction for β-actin confirms similar loading of protein. (B) Quantitative estimation of proteins in (A) after normalization with β-actin shows a decrease in ferritin, and an increase in Tf and TfR levels in PrP^KO^ samples compared to Wt controls. Values are mean±SEM. *p<0.001, **p<0.01 relative to Wt. n = 6.

Since PrP is expressed most abundantly on neurons, the effect of PrP deletion on brain iron homeostasis was assessed in PrP^KO^ and matched Wt controls. In iron deficiency, we expect low levels of total iron, decreased levels of iron storage protein ferritin, and increased levels of iron uptake proteins Tf and TfR in the affected tissue. To evaluate these parameters, equal quantity of protein from brain homogenates of PrP^KO^ and Wt mice was fractionated by SDS-PAGE and immunoblotted (Figure 1A). Probing for PrP reveals the expected glycoforms migrating between 27–37 kDa in Wt, and complete absence of PrP in PrP^KO^ samples as expected. Probing for ferritin shows a decrease in H- and L-chain isoforms in PrP^KO^ samples relative to Wt controls. Tf and TfR, on the other hand, show a relative increase in PrP^KO^ samples. Probing for β-actin confirms that the observed differences are not an artifact of protein loading (Figure 1A, lanes 1–6). Quantification by densitometry shows a decrease in ferritin levels by 59% and an increase in Tf and TfR levels by 58% and 245% respectively in PrP^KO^ samples relative to Wt controls (Figure 1B).

**Figure 2:**
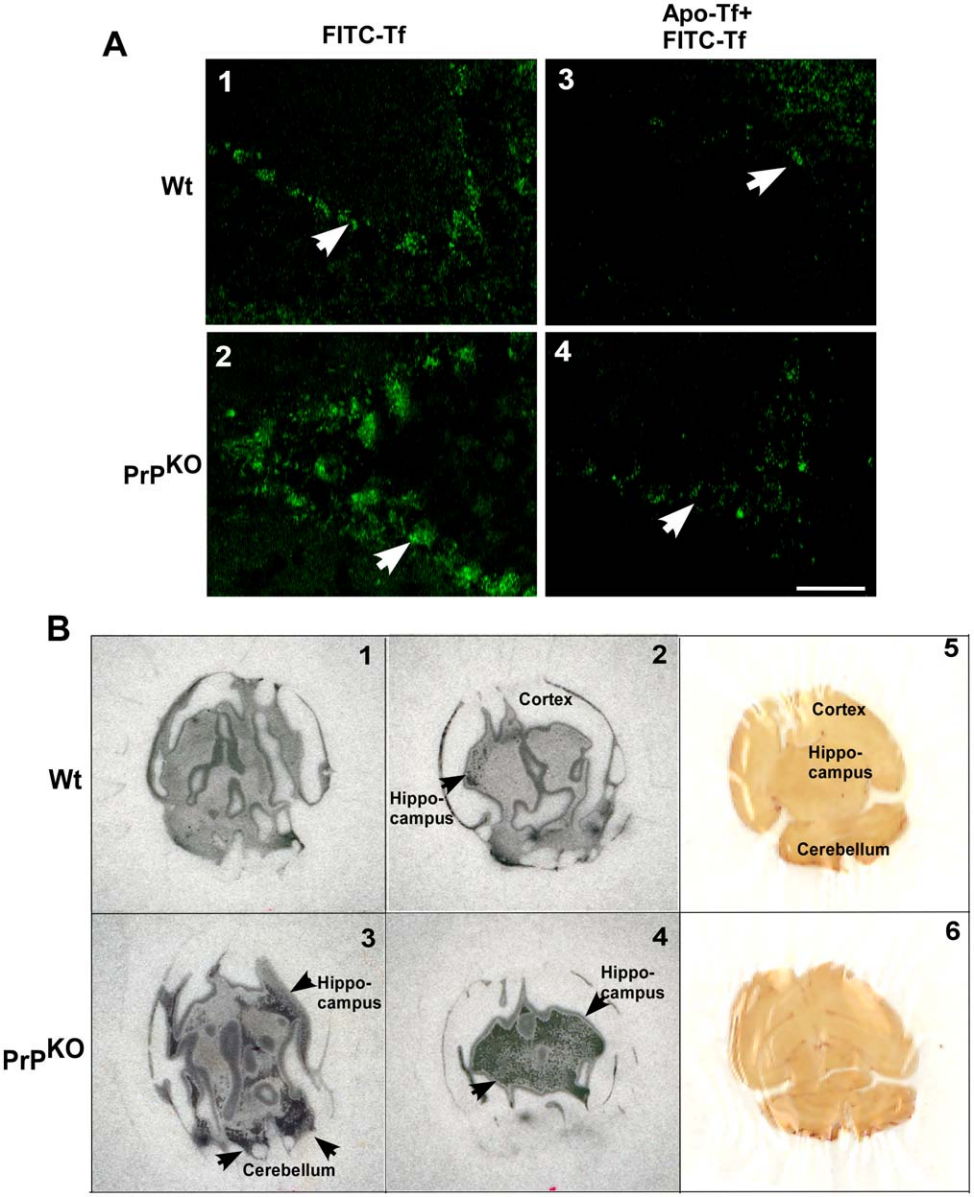
Brain sections of PrP^KO^ mice show up regulation of TfR and increased transport of iron from the blood stream. (A) Incubation of unfixed brain sections with FITC-Tf shows significantly more binding to the Purkinje cell neurons of PrP^KO^ sample compared to Wt controls (panels 1 and 2) [16]. Pre-incubation of brain sections with unlabeled apo-Tf decreases the signal significantly in both samples (panels 3 and 4), demonstrating the specificity of binding. The assay was performed on three sections each from three different sets of mice. A representative section is shown. (B) Autoradiograph of brain sections prepared from Wt and PrP^KO^ mice injected with ^59^FeCl_3_ intravenously shows significantly more ^59^Fe in PrP^KO^ sections compared to Wt controls (panels 1–4). A scan of brain sections exposed to the film in panels 2 and 4 respectively is shown (panels 5 and 6). (Two consecutive 700 µM sections from each brain sample are shown. ^59^Fe is visible as radio-opaque black dots.

Brain homogenate represents a mixture of different cell types and the above results do not necessarily reflect the iron status of neurons, the cell population most vulnerable to alterations in iron homeostasis. To evaluate the iron status of neurons, unfixed brain sections from Wt and PrP^KO^ mice were incubated with FITC-tagged Tf for 15 minutes at 37°C [16], washed gently with cold PBS to remove unbound Tf, and fixed with paraformaldehyde. A set of control samples were pre-incubated with unlabeled apo-Tf for 15 minutes before exposure to FITC-Tf to compete for binding sites. Examination by fluorescence microscopy shows significantly more binding of FITC-Tf to the cerebellar Purkinje cell layer of PrP^KO^ brain sections compared to Wt controls, suggesting higher expression of TfR in PrP^KO^ neurons (Figure 2A, panels 1 and 2, white arrow). The binding of FITC-Tf is inhibited competitively by pre-incubation of brain sections with unlabeled apo-Tf, indicating the specificity of the reaction (Figure 2A, panels 3 and 4, white arrow-head). To further analyze the state of iron deficiency in PrP^KO^ brains, equal counts of ^59^FeCl_3_ were injected in the tail vein of Wt and PrP^KO^ mice, and after a chase of 3 hours, mice were euthanized and cold PBS was infused from the heart to drain capillary blood. Subsequently, brains were harvested, counted in a γ-counter, and fixed overnight in formalin. Subsequently, 700 µM sections were cut and exposed to X-ray film. Brains of PrP^KO^ mice take up 45% more ^59^Fe relative to Wt controls as estimated by total counts and visualization on X-ray film exposed to dry sections (Figure 2B, panels 1–4, black arrows). These results indicate a state of relative iron deficiency and consequent increased transport of ^59^Fe to the brains of PrP^KO^ mice.

**Figure 3:**
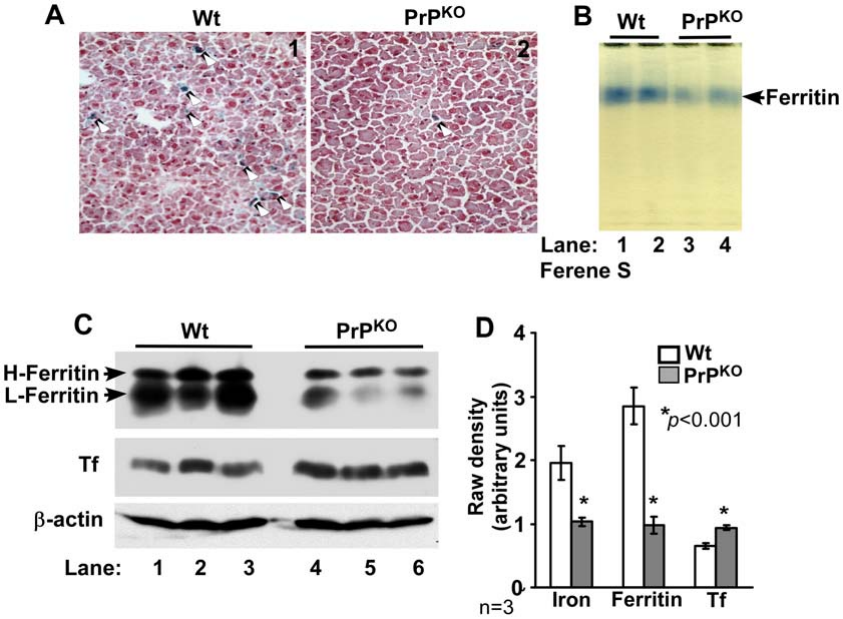
Liver of PrP^KO^ mice displays a phenotype of iron deficiency. (A) Prussian blue staining of frozen sections shows minimal reaction for iron in the PrP^KO^ sample compared to Wt controls. (B) In-gel reaction of homogenates with Ferene-S shows significantly less iron content of PrP^KO^ ferritin compared to Wt controls (lanes 1–4). (C) Immunoreaction of homogenates with anti-ferritin antibody specific for H and L chains shows significantly less ferritin expression in PrP^KO^ samples compared to Wt controls (lanes 1–6). Re-probing for Tf shows an increase in Tf expression in PrP^KO^ samples relative to Wt controls (lanes 1–6). Levels of β-actin are similar in all samples, confirming that the observed differences are not an artifact of protein loading (lanes 1–6). (D) Quantification by densitometry shows a significant reduction in the iron content and expression of ferritin, and an increase in Tf expression in PrP^KO^ samples compared to matched Wt controls. Values are mean±SEM, n = 3. *p<0.001 relative to Wt.

To determine whether a similar phenotype of iron deficiency is observed in other major organs of PrP^KO^ mice, the iron content and levels of the iron management proteins ferritin and Tf were evaluated in the liver of Wt and PrP^KO^ mice. Frozen sections of Wt and PrP^KO^ liver were stained for iron with Prussian blue and examined. PrP^KO^ sections show significantly less iron in Kupffer cells and hepatocytes relative to Wt controls (Figure 3A, panels 1 and 2). Fractionation of liver homogenates on a non-denaturing gel followed by in-gel reaction with Ferene-S shows lower iron content in PrP^KO^ liver ferritin compared to Wt controls (Figure 3B, lanes 1–4) [17]. (The identity of the blue band as ferritin was established by immunoblotting as described previously [13]). Evaluation of ferritin and Tf expression in liver homogenates shows significantly lower levels of ferritin and higher levels of Tf in PrP^KO^ samples compared to Wt controls (Figure 3C, lanes 1–6). Quantification by densitometry shows a decrease in the iron content and expression of ferritin by 48% and 66% respectively, and an increase in the expression level of Tf by 44% in PrP^KO^ samples relative to Wt controls (Figure 3D).

**Figure 4:**
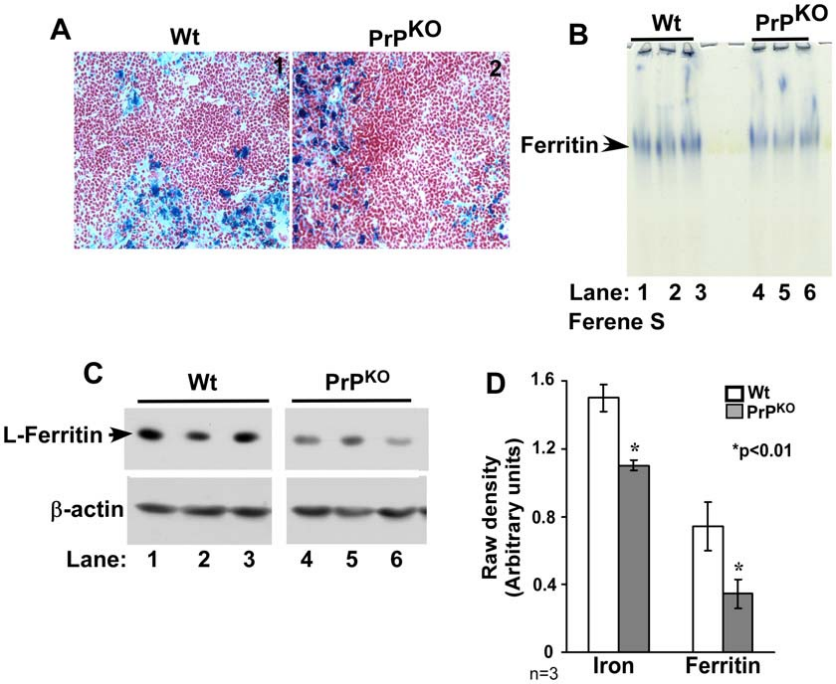
Spleen of PrP^KO^ mice displays a phenotype of iron deficiency. (A) Prussian blue staining of frozen sections shows a prominent reaction in the reticulo-endothelial cells of PrP^KO^ and Wt samples. (B) In-gel reaction of homogenates with Ferene-S shows less iron content of PrP^KO^ ferritin compared to Wt controls (lanes 1–6). (C) Immunoreaction of homogenates with L-chain specific anti-ferritin antibody shows significantly less expression in PrP^KO^ samples compared to Wt controls (lanes 1–6). Re-probing for β-actin confirms that the difference is not an artifact of protein loading (lanes 1–6). (D) Quantification by densitometry after normalization with β-actin shows a significant reduction in the iron content and expression of ferritin in PrP^KO^ samples compared to matched Wt controls. Values are mean±SEM, n = 3. *p<0.01 relative to Wt.

A similar analysis of spleen sections shows intense reaction for iron with the Perl's stain in both Wt and PrP^KO^ samples (Figure 4A, panels 1 and 2). In-gel reaction of spleen ferritin with Ferene-S shows a decrease in the iron content and expression of ferritin in PrP^KO^ samples relative to Wt controls (Figures 4B and C, lanes 1–6). Quantification by densitometry shows a decrease in the iron content and expression of ferritin by 27%, and 64% in PrP^KO^ samples compared to Wt controls (Figure 4D). Re-probing of the membrane for Tf and TfR did not show a significant difference in the expression of these proteins (data not shown).

Together, the above results suggest a phenotype of relative iron deficiency in the brain, liver, and spleen of PrP^KO^ mice compared to Wt controls. Subsequent studies were directed at characterizing the iron deficient phenotype of PrP^KO^ mice and the mechanistic basis of this finding.

### The iron deficiency of PrP^KO^ mice is largely compensated

To evaluate if the phenotype of iron deficiency in PrP^KO^ mice is due to low circulating iron, hematological parameters were evaluated by an automated blood chemistry analyzer optimized for mouse samples. Hemoglobin, serum iron, Tf saturation, and serum ferritin are decreased in PrP^KO^ samples by 3.5, 21.6, 37.6 and 50.1% respectively, while the reticulocyte count and total iron binding capacity (TIBC) are increased by 42.2 and 24.0% respectively in PrP^KO^ samples relative to matched Wt controls (Table 1). The total red cell count and hematocrit show minimal differences between the two groups. Bone marrow smears revealed an increase in red cell precursors in PrP^KO^ samples, indicating an attempt by the iron homeostatic machinery to compensate for the iron deficiency in PrP^KO^ animals (data not shown).

**Table 1:** Hematological parameters in Wt and PrP^KO^ mice.

	Wt	PrP^KO^	% change in PrP^KO^	N
**RBC (10^12^/L)**	9.23±0.12	9.20±0.33	NS	14
**Hematocrit (%)**	49.01±1.08	49.05±1.45	NS	14
**Hemoglobin (g/dl)**	14.54±0.19	14.02±0.28***	↓ 3.5	14
**Reticulocytes (%)**	3.46±0.23	4.92±0.27*	42.2↑	10
**Serum iron (µg/dl)**	325.0±6.91	254.5±14.8*	↓ 21.6	6
**TIBC (µg/dl)**	451.73±25.8	561.09±36.61**	24.0 ↑	6
**Tf saturation (%)**	70.31±4.52	43.86±6.12**	↓ 37.6	6
**Serum ferritin (ng/dl)**	98.51±4.82	49.13±6.43*	↓ 50.1	6

Values are mean±SEM. ^*^ *p*<0.001, ^**^ *p*<0.01, ^***^ *p*<0.05 as compared to Wt. NS: not significant; N: number of animals in each group; RBCs: red blood cells; TIBC: total iron binding capacity; Tf: transferrin; ↓: decrease; ↑: increase.

Subsequent studies were directed at determining whether the iron deficiency in PrP^KO^ mice arises from inefficient uptake and/or transport of iron from ingested food, defective uptake by target cells of different organ systems, or a combination of both processes.

### PrP mediates iron uptake and/or transport from the duodenum to the blood stream

**Figure 5:**
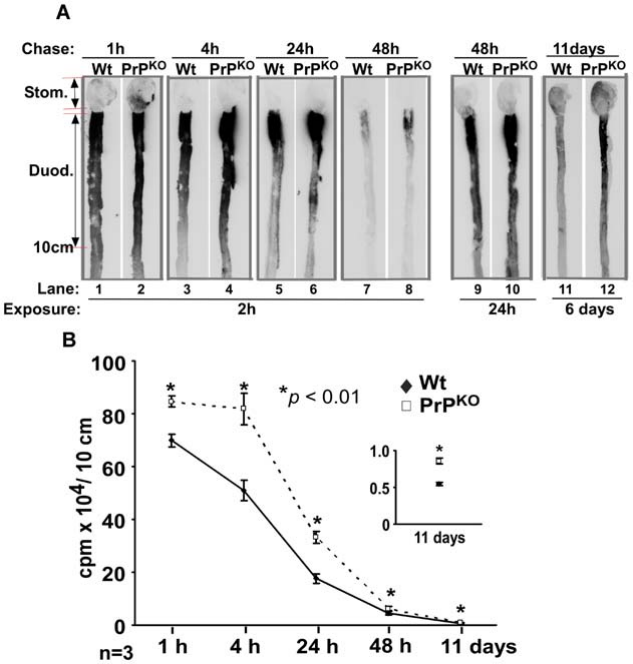
Iron is retained in the duodenum of PrP^KO^ mice. (A) Autoradiogram of upper gastrointestinal tract of Wt and PrP^KO^ mice fed 20 µCi of ^59^FeCl_3_ by gastric gavage shows higher levels of ^59^Fe in PrP^KO^ samples after 1, 4, 24, and 48 hours of chase (lanes 1–10). The difference becomes more obvious after 11 days of chase (lanes 11 and 12). (Lanes 9 and 10 were exposed for 24 hours and lanes 11 and 12 for 6 days to observe the difference clearly). A representative experiment out of a total of three is shown. (B) Quantification of ^59^Fe in the first 10 cm of the duodenum shows significantly higher ^59^Fe in the PrP^KO^ samples at all time points tested compared to matched Wt controls. Values are mean±SEM, n = 3. *p<0.01 as compared to Wt.

The uptake and transport of ingested iron from the intestinal lumen to the blood stream and target organs of Wt and PrP^KO^ mice was evaluated by introducing radioactive iron into the stomach, and quantifying ^59^Fe uptake by various organs after defined time points of chase. The following organs were evaluated: 1) stomach with attached duodenum, jejunum, and a small piece of ileum, 2) liver, 3) spleen, 4) brain, 5) femurs, and 6) tibial bones. Washed upper gastrointestinal tract was vacuum-dried and exposed to X-ray film (Figure 5A), and after obtaining adequate film exposures, ^59^Fe incorporated in the upper 10 cm of the duodenum was quantified in a γ-counter. Whole blood, washed red blood cells (RBCs), plasma, organs, and bones were counted in a γ-counter to quantify ^59^Fe incorporation.

Autoradiographs of duodenum samples demonstrate significantly more ^59^Fe in PrP^KO^ samples at each time point compared to Wt controls (Figure 5A, lanes 1–12). Surprisingly, the amount of ^59^Fe in the duodenum of PrP^KO^ mice is significantly higher than Wt samples even after 11 days of chase (Figure 5A, lanes 11 and 12). Samples in lanes 1–8 were exposed to X-ray film for 2 hours, lanes 9 and 10 for 24 hours, and lanes 11 and 12 for 6 days to highlight the difference in ^59^Fe content between Wt and PrP^KO^ samples (Figure 5A, lanes 9–12). Quantitative analysis of these results was performed by counting the first 10 cm of the duodenum from each sample in a γ-counter. As expected, both samples show a gradual decline in ^59^Fe counts with increasing chase time, falling to 0.1% of the initial value after 11 days of chase. However, PrP^KO^ samples show higher retention of ^59^Fe by 21, 60, 87, 32, and 57% relative to matched Wt controls after a chase of 1, 4, 24, and 48 hours and 11 days respectively (Figure 5B). Mice ranging in age from 3–6 months and blinded to the person performing the experiment yielded similar results. The 1 and 4 hour chase time points were repeated more than 6 times.

**Figure 6:**
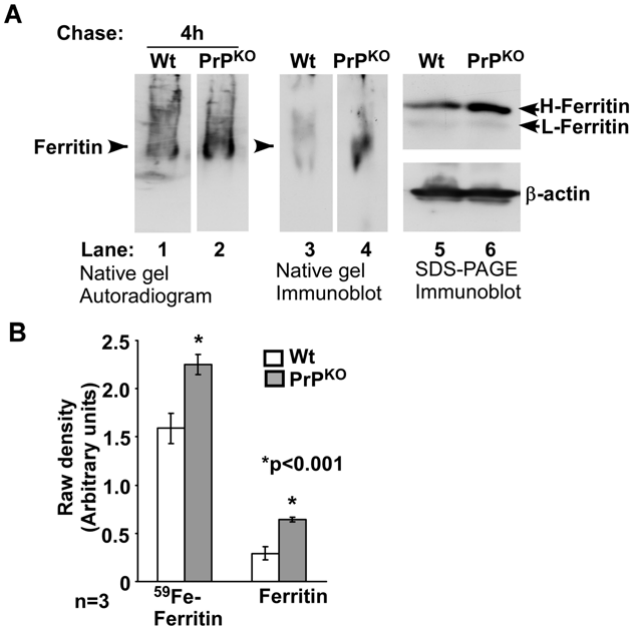
Iron is retained within ferritin in the duodenal epithelium of PrP^KO^ mice. (A) The 4 hour duodenum samples in Figure 5 above were homogenized and separated on a non-denaturing gel followed by autoradiography (lanes 1 and 2). The level of ^59^Fe labeled ferritin is significantly higher in the PrP^KO^ sample compared to matched Wt control (lanes 1 and 2). Transblotting under native conditions followed by immunoblotting for ferritin confirms the identity of ^59^Fe labeled bands as ferritin (lanes 3 and 4). Fractionation of the same samples by SDS-PAGE followed by immunoblotting shows relatively more ferritin in the PrP^KO^ sample compared to matched Wt control (lanes 5 and 6). (B) Quantification by densitometry shows significantly more ^59^Fe-ferritin and ferritin protein levels in the duodenum of PrP^KO^ mice relative to matched Wt controls. Values are mean±SEM, n = 3. *p<0.001 relative to Wt.

To evaluate whether increased retention of ^59^Fe in the duodenum of PrP^KO^ mice is due to sequestration in ferritin within enterocytes, ^59^FeCl_3_ fed Wt and PrP^KO^ mice were chased for 4 hours, and the first 5 cm of the duodenum was homogenized and fractionated on a non-denaturing gel to identify ^59^Fe-labeled proteins as described previously [13]. A single iron labeled band that immunoreacts for ferritin is detected in Wt and PrP^KO^ samples (Figure 6A, lanes 1–4). The iron content and expression of ferritin are higher in the PrP^KO^ sample compared to Wt control, explaining the significantly higher ^59^Fe content in the duodenum of PrP^KO^ mice (Figure 6A, lanes 1–4). Fractionation of the same samples by SDS-PAGE followed by immunoblotting confirms that ferritin levels are significantly higher in the PrP^KO^ sample (Figure 6A, lanes 5 and 6). (The identity of iron labeled bands in lanes 1 and 2 was confirmed by eluting labeled proteins from these bands and re-fractionating on SDS-PAGE followed by immunoblotting as described previously [13]). Quantification by γ-counting and densitometry shows an increase in the ^59^Fe content of ferritin by 42% and expression of ferritin by 119% in PrP^KO^ samples relative to matched Wt controls (Figure 6B).

These results indicate that PrP^KO^ mice take up more ^59^Fe from the intestinal lumen and/or release less into the blood stream compared to Wt controls, resulting in the accumulation of ^59^Fe in the duodenal epithelium.

### PrP facilitates iron uptake by parenchymal cells and hematopoietic precursor cells

**Figure 7:**
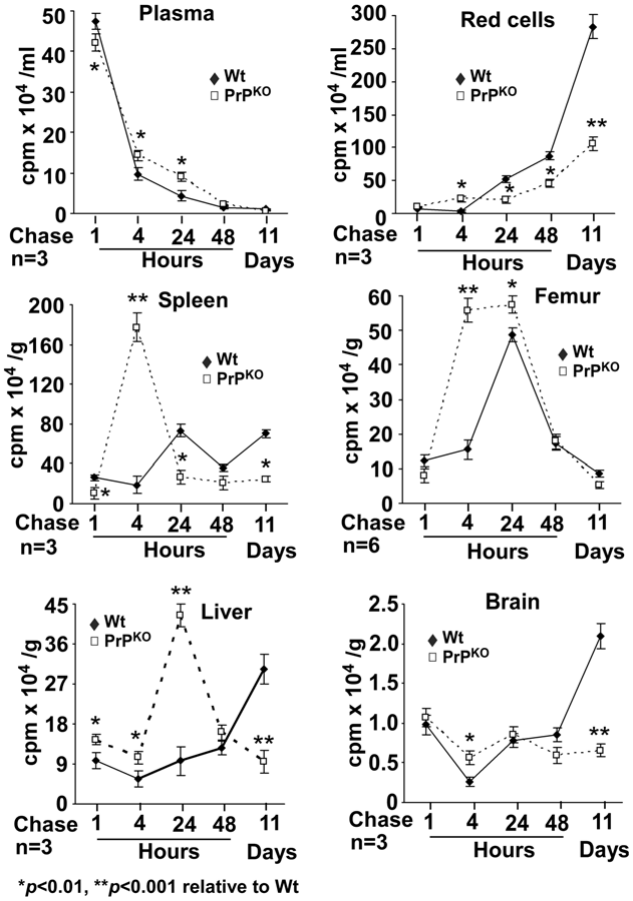
Distribution of ^59^Fe in Wt and PrP^KO^ mice. Plasma: ^59^Fe in PrP^KO^ samples is lower after 1 hour, increases by 48 hours, and falls to values below Wt after 11 days of chase. RBCs: Wt RBCs show ^59^Fe incorporation after 24 hours, and the amount increases steadily till 11 days of chase. PrP^KO^ RBCs, on the other hand, incorporate ^59^Fe relatively sooner after 4 hours, and show only a modest increase till 11 days of chase. Except for the 4 hour chase time, the amount of ^59^Fe in PrP^KO^ RBCs is significantly lower than Wt controls, especially after 11 days of chase. Spleen: Wt samples show a slight increase in ^59^Fe after 24 hours, and minimal change thereafter. PrP^KO^ spleens, on the other hand, show a significant increase after 4 hours, followed by a precipitous fall after 24 hours. Femur: Kinetics of ^59^Fe uptake mirrors that of the spleen. PrP^KO^ samples show a spike at 4 hours that is maintained till 24 hours, followed by a fall. Wt samples show a delayed peak at 24 hours, and a fall to similar levels as the PrP^KO^ sample after 11 days. Liver: Wt samples show a steady increase in ^59^Fe till 11 days of chase. PrP^KO^ samples, on the other hand, show a peak at 24 hours, and the counts fall thereafter to values significantly lower than Wt controls. Brain: Wt and PrP^KO^ samples do not show much difference at early time points of chase, but ^59^Fe incorporation in the PrP^KO^ sample is significantly lower than Wt controls after 11 days of chase. Values are mean±SEM of three independent experiments. *p<0.01, **p<0.001 relative to Wt.

Transport of iron from the duodenal epithelium to the blood stream and subsequent uptake by various organs was evaluated by quantifying ^59^Fe in the plasma, washed RBCs, and various organs in a γ-counter, and fractionating the plasma using a non-denaturing gel to evaluate the ^59^Fe content of Tf. The ^59^Fe counts in the plasma of PrP^KO^ mice are decreased by 12% after 1 hour, and increased by 47, 108, 51, and 40% after 4, 24, and 48 hours and 11 days of chase respectively relative to Wt controls (Figure 7, plasma). Red blood cells show no incorporation of ^59^Fe after 1 hour as expected, a sudden increase in PrP^KO^ samples by 537% after 4 hours, and a decrease by 60, 48, and 63% after 24 and 48 hours and 11 days respectively relative to Wt controls (Figure 7, red cells). The spleen of PrP^KO^ mice shows 62% less ^59^Fe after 1 hour, an increase by 831% after 4 hours, followed by a precipitous fall by 64% of Wt values after 24 hours that is maintained throughout 11 days of chase (Figure 7, spleen). Likewise, the femurs of PrP^KO^ mice show 25% less ^59^Fe after 1 hour, an increase by 261 and 17% after 4 and 24 hours, and a decline by 39% after 11 days respectively relative to Wt controls. Femurs of Wt mice also show a spike in ^59^Fe after 24 hours of chase, but the increase is significantly less than PrP^KO^ samples (Figure 7, Femur). The liver of PrP^KO^ mice shows an increase in ^59^Fe by 47 and 82% after 1 and 4 hours, an increase by 339% after 24 hours, and a precipitous fall by 69% after 11 days of chase relative to Wt controls (Figure 7, liver). Brain samples show variability during the first 48 hours of chase in both PrP^KO^ and Wt mice, but after 11 days there is a significant decrease in PrP^KO^ samples by 69% of Wt values (Figure 7, brain).

**Figure 8:**
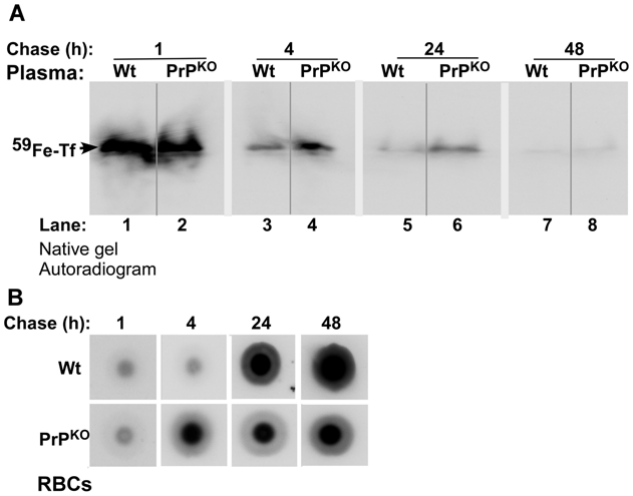
Time course of ^59^Fe distribution in the blood of Wt and PrP^KO^ mice. (A): Equal volume Wt and PrP^KO^ plasma collected at different time points of chase were separated on a non-denaturing gel followed by autoradiography. After 1 hour of chase, ^59^Fe-Tf in the PrP^KO^ sample is lower than matched Wt controls (lanes 1 and 2). However, the ratio reverses thereafter, and the level of ^59^Fe-Tf in the PrP^KO^ sample is higher than Wt controls after 4, 24, and 48 hours of chase (lanes 3–8). (B) RBCs of Wt mice show minimal levels of ^59^Fe after 1 and 4 hours, and significant increase after 24 and 48 hours of chase. PrP^KO^ mice, on the other hand, show iron counts at 4 hours, and minimal increase thereafter.

Fractionation of plasma by non-denaturing gel electrophoresis followed by autoradiography reveals the presence of ^59^Fe-Tf in Wt and PrP^KO^ samples as expected (Figure 8A) (The identity of ^59^Fe labeled band as Tf was established by immunoblotting as reported earlier [13]). The ^59^Fe content of Tf in the PrP^KO^ sample is lower after 1 hour and significantly higher after 4 and 24 hours of chase relative to Wt controls (Figure 8A, lanes 1–6). Minimal signal is detected after 48 hours of chase in either of the samples (Figure 8A, lanes 7 and 8). Evaluation of the ^59^Fe content of RBCs spotted on a PVDF membrane followed by autoradiography shows increased incorporation in PrP^KO^ samples after 4 hours and minimal increase thereafter until 48 hours of chase. Wt samples, on the other hand, show significant incorporation after 24 hours and almost doubling of the counts by 48 hours (Figure 8B), reflecting the values in Figure 7 above. These results indicate inefficient transport of ^59^Fe from the duodenum of PrP^KO^ mice to the blood stream, but much faster kinetics of incorporation into RBC precursors by 4 hours of chase and minimal increase thereafter. In contrast, Wt mice show significantly higher transport of ^59^Fe to the blood stream, incorporation into RBCs at the expected chase time of 24 hours, and a linear increase in the ^59^Fe content of RBCs thereafter. Since the majority of Tf-bound ^59^Fe is taken up by newly synthesized RBCs, the increase in plasma ^59^Fe-Tf in PrP^KO^ mice after 4 hours of chase relative to Wt controls indicates decreased uptake as evidenced by the lower ^59^Fe content of PrP^KO^ RBCs (Figure 8A, lanes 3–6).

Together, the above results indicate that the transport of iron from the duodenal enterocytes to the blood stream and uptake by the RBCs, liver, and brain is less efficient in PrP^KO^ mice and follows different kinetics of utilization relative to Wt controls. It is likely that PrP^KO^ mice make an early attempt at erythropoiesis because of their iron deficient state, but are unable to incorporate enough iron into RBCs due to defective uptake by hematopoietic progenitor cells. The lower ^59^Fe content of PrP^KO^ RBCs is not due to a shorter life-span of these cells since their ^59^Fe counts remain constant throughout 11 days of chase (Figure 7), the normal half-life of murine RBCs. No signs of hemolysis were detected at any time point of chase by urine-analysis (data not shown).

### Iron deficient phenotype of PrP^KO^ mice is not due to sequestration of iron in reticulo-endothelial cells

**Figure 9:**
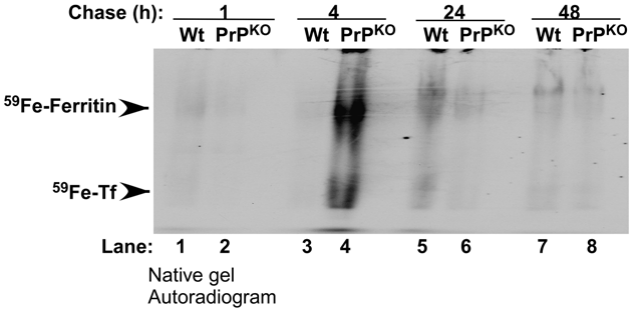
Kinetics of ^59^Fe uptake by Wt and PrP^KO^ spleen. Autoradiogram of Wt and PrP^KO^ spleen homogenates separated on a native gel show significantly higher ^59^Fe-ferritin and ^59^Fe-Tf in the PrP^KO^ sample after 4 hours of chase (lanes 3 and 4). At other time points Wt samples show higher levels of ^59^Fe in ferritin and Tf bands relative to PrP^KO^ samples (lanes 1 and 2 and 5–8). (The identity of ferritin and Tf bands was confirmed by immunoblotting [13]).

To rule out low level of hemolysis combined with sequestration of ^59^Fe in cells of the reticulo-endothelial system in PrP^KO^ mice, spleens harvested after 1–48 hours of chase (as in Figure 7 above) were homogenized, and equal quantity of protein was fractionated by non-denaturing gel electrophoresis followed by autoradiography. Prominent bands of ^59^Fe-ferritin and ^59^Fe-Tf are detected in the PrP^KO^ sample after 4 hours of chase revealing ^59^Fe content several-fold higher than matched Wt controls (Figure 9, lanes 3 and 4). However, the signal is lost by 24 hours of chase, making it unlikely that reticulo-endothelial cells sequester the incorporated ^59^Fe (Figure 9, lanes 5–8). At all other time points the Wt sample shows slightly higher ^59^Fe-ferritin and ^59^Fe-Tf compared to PrP^KO^ samples (Figure 9, lanes 1, 2, and 5–8). The identity of ^59^Fe labeled bands as ferritin and Tf was established by immunoblotting under native conditions as described previously [13].

Further confirmation of the above results was obtained by culturing peritoneal macrophages from Wt and PrP^KO^ mice overnight, loading them with ^59^FeCl_3_-citrate complex after stimulation with LPS [18], and monitoring release of ^59^Fe into the medium in a γ-counter. Macrophages from PrP^KO^ mice release 17% of intracellular iron into the medium compared to 13% from the Wt sample during a chase of 18 hours, making it unlikely that sequestration of ^59^Fe in the reticulo-endothelial cells is responsible for the iron deficient phenotype of PrP^KO^ mice (Table 2).

**Table 2:** Release of ^59^Fe from peritoneal macrophages after 18 hours.

	Intracellular	Secreted (18 h)	% Secreted
**Wt**	151689±12376	20311±328	13%
**PrP^KO^**	141413±1995	24054±604*	17%

Values are mean±SEM of cells plated in triplicate from two different sets of mice. ^*^ *p*<0.01 compared to Wt.

### Expression of wild type PrP rescues the iron deficient phenotype of PrP^KO^ mice

**Figure 10:**
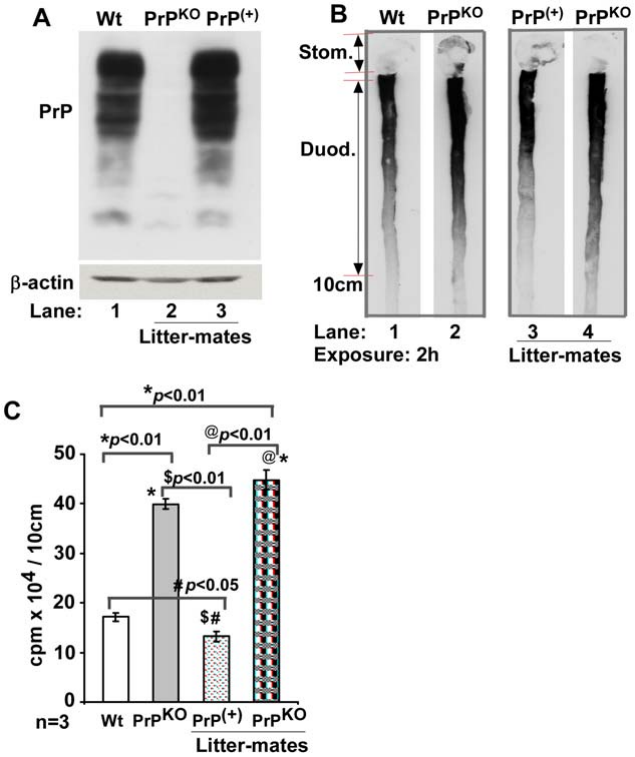
Iron deficiency in PrP^KO^ mice is reversed by expressing Wt PrP. (A) Immunoblot analysis of Wt and littermate PrP^KO^ and PrP^(+)^ brain homogenates for PrP shows the expected glycoforms of PrP in Wt and PrP^(+)^ samples (lanes 1 and 3), and no reaction in the PrP^KO^ sample as expected (lane 2). (B) Autoradiogram of the upper gastrointestinal tract of Wt and littermate PrP^(+)^ and PrP^KO^ mice fed ^59^FeCl_3_ by gastric gavage as above and chased for 4 hours shows significantly more ^59^Fe in the PrP^KO^ sample relative to Wt control (lanes 2 and 4). Notably, the ^59^Fe content of PrP^(+)^ duodenum is similar to Wt, and significantly less than the littermate PrP^KO^ sample (lanes 1, 3 and 4). (C) Quantification of ^59^Fe in the first 10 cm of the duodenum shows significantly more ^59^Fe in the PrP^KO^ sample compared to matched Wt control. Similarly, more ^59^Fe is detected in the PrP^KO^ sample compared littermate PrP^(+)^ control. One way ANOVA shows a significant difference in ^59^Fe incorporation between Wt and PrP^KO^, Wt and littermate PrP^KO^, PrP^(+)^ and littermate PrP^KO^, and PrP^KO^ and PrP^(+)^ (*p*<0.0001). Post hoc Bonferroni tests show significant difference in ^59^Fe in PrP^KO^ as well as littermate PrP^(+)^ and PrP^KO^ duodenums compared to Wt control (*p<0.01; #*p*<0.05). PrP^(+)^ exhibit significant difference compared to PrP^KO^ ($*p*<0.01), and littermate PrP^(+)^ and PrP^KO^ also differ significantly (@p<0.01). Values are mean±SEM, n = 3.

Since the only difference between Wt and PrP^KO^ mice is the absence of PrP expression in the latter, a single allele of Wt PrP was re-introduced on the PrP^KO^ background to generate PrP^+^ mice. To assess the expression levels of PrP in PrP^+^ mice relative to Wt controls, an equal quantity of protein from brain homogenates of Wt, PrP^KO^, and PrP^+^ mice was fractionated by SDS-PAGE and immunoblotted. Probing for PrP shows the expected glycoforms of PrP migrating between 27–37 kDa in Wt and PrP^+^ samples (Figure 10A, lanes 1 and 3). Quantification by densitometry shows slightly higher PrP expression in PrP^+^ mice, ∼10% relative to Wt controls. PrP^KO^ samples do not react for PrP as expected (Figure 10A, lane 2). Thus, PrP^+^ mice are a suitable model to test whether expression of PrP rescues the iron deficient phenotype of PrP^KO^ mice.

To reduce variability, littermate PrP^KO^ and PrP^+^ mice were obtained by crossing PrP^+^ and PrP^KO^ breeding pairs. After confirming the genotype (data not shown), littermate PrP^+^ and PrP^KO^, Wt, and PrP^KO^ mice were evaluated for iron uptake and transport by introducing equal amounts of ^59^FeCl_3_ by gastric gavage as in Figures 5 and 7 above (Figure 10B and C). Only the 4 hour chase time point was assessed since the maximum differences between Wt and PrP^KO^ mice are observed at this time point (Figure 5 and 7 above). Autoradiography of duodenum samples shows significantly more ^59^Fe in the PrP^KO^ sample compared to the Wt control as noted in Figure 5 above (Figure 10B, lanes 1 and 2). Notably, a similar difference is seen between PrP^KO^ and PrP^+^ littermates (Figure 10B, lanes 3 and 4), indicating improved transport of ^59^Fe from the duodenum of mice expressing PrP. Wt and PrP^+^ samples show similar levels of iron, confirming the above results (Figure 10A, lanes 1 and 3).

**Figure 11:**
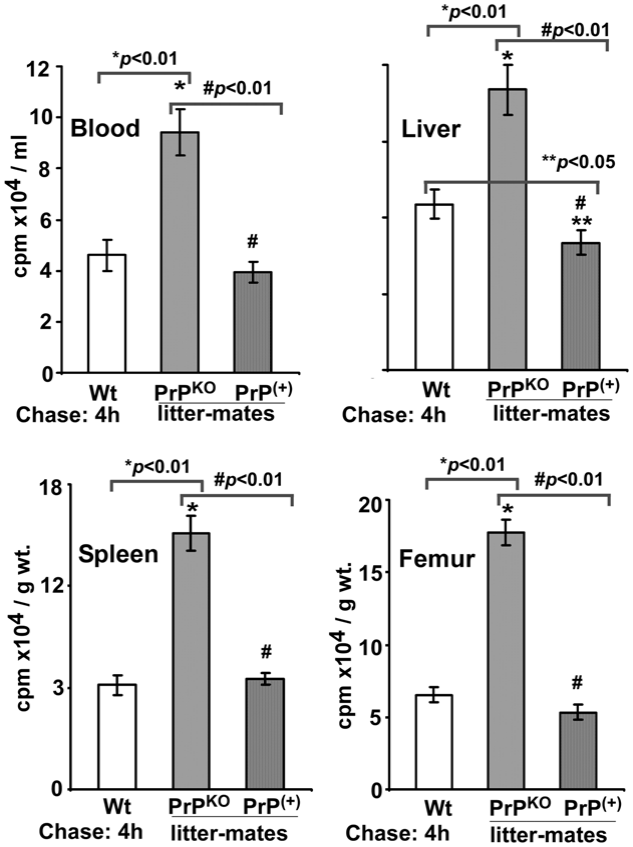
Distribution of ^59^Fe in PrP^(+)^ and PrP^KO^ litter-mates. Distribution of ^59^Fe in the blood, liver, spleen, and femur after 4 hours of chase shows similar incorporation in Wt and PrP^(+)^ mice in the organs tested, and significantly more ^59^Fe in the spleen and femur of PrP^KO^ mice as noted above. One way ANOVA shows a significant difference among Wt, PrP^KO^, and PrP^(+)^ samples (p<0.0001). Post hoc Bonferroni tests show significant difference in ^59^Fe in littermates PrP^(+)^ and PrP^KO^ organs relative to Wt controls (*p<0.01; **p<0.05). Littermate PrP^(+)^ shows a significant difference compared to PrP^KO^ counterpart (#p<0.01). Values are mean±SEM of three independent experiments.

Quantification of ^59^Fe in the first 10 cm of duodenum samples shows an increase in PrP^KO^ samples by 132% relative to Wt controls, and an increase in littermate PrP^KO^ by 238% relative to littermate PrP^+^ controls (Figure 10C). As expected, Wt and PrP^+^ samples show minimal difference in their ^59^Fe content (Figure 10C, lanes 1 and 3). Quantification of ^59^Fe in the blood, liver, spleen and femur shows similar incorporation in Wt and PrP^+^ controls, and higher levels in PrP^KO^ mice as observed in Figure 7 above (Figure 11). These observations demonstrate that the deficiency of iron uptake in PrP^KO^ mice is reversed by the expression of PrP in the PrP^+^ mice.

## Discussion

In this report, we demonstrate that the absence of PrP induces systemic iron deficiency in PrP^KO^ mice, a phenotype that is rescued by re-introducing PrP on the PrP^KO^ background. Relative to normal Wt mice of the same genetic background, PrP^KO^ mice exhibit lower levels of iron in the plasma, brain, liver, and spleen, and the major iron metabolism proteins show a compensatory response by increasing the levels of iron uptake proteins Tf and TfR, and decreasing the levels of iron storage protein ferritin. The underlying cause of iron deficiency in PrP^KO^ mice is likely to be two-fold; 1) inefficient transport from duodenal enterocytes to the blood stream, and 2) impaired uptake by target cells of various organs, including the hematopoietic precursor cells. We previously reported that PrP functions as an iron uptake and transport protein in human neuroblastoma cells *in vitro* [13]. This report supports and extends those findings by demonstrating a similar function of PrP in mouse models, a novel function for a protein known mostly for its role in the pathogenesis of prion disorders.

It is surprising that the mere absence of PrP alters iron metabolism in both enterocytes and hematopoietic cells since distinct processes regulate iron uptake and transport at these sites. In the intestine, absorption of non-heme iron, the form used in this study, is mediated by the divalent metal transporter 1 (DMT1) at the apical (AP) plasma membrane of duodenal epithelial cells. Here, duodenal cytochrome b (DcytB) functions as a ferric reductase and reduces Fe(III) iron to the Fe(II) form for transport to the cytosol by DMT1. Absorbed iron is transported through the cytosol by poorly characterized mechanisms and is exported from the basolateral (BL) membrane by the concerted effort of ferroportin (FPT) and hephaestin, a multicopper oxidase that oxidizes Fe(II) iron to the Fe(III) form for uptake by Tf in the systemic circulation [19].

Based on our observations, it appears that PrP mediates iron export from the BL membrane of enterocytes to the blood stream rather than uptake from the intestinal lumen. This conclusion is supported by our observations demonstrating accumulation of orally administered radioactive iron (^59^FeCl_3_) in the duodenal enterocytes of PrP^KO^ mice that persists until eleven days of chase when almost all ^59^Fe from Wt controls has been transported out. Most of the ^59^Fe accumulates in ferritin within these cells, a surprising observation given the state of iron deficiency in these mice. Ferritin protein expression is also up-regulated in PrP^KO^ enterocytes, probably reflecting a response to increased iron influx from the AP plasma membrane, decreased transport from the BL membrane, or a combination of the two processes. Although limited amounts of ^59^Fe are released into the blood stream of PrP^KO^ mice during the first hour of chase, the amount is significantly lower compared to Wt controls. Furthermore, PrP is expressed on the BL surface of MDCK cells, a polarized epithelial cell line, and inter-cellular junctions of mouse enterocytes [20], [21], suggesting a functional role at the BL domain rather than the AP plasma membrane. It is surprising that PrP^KO^ mice excrete relatively less ^59^Fe in their feces despite the iron loaded state of their enterocytes (unpublished observations). It is likely that this response reflects an attempt to conserve and utilize the absorbed iron by PrP^KO^ mice due to their chronic state of iron deficiency.

A similar phenotype of systemic iron deficiency resulting from accumulation of absorbed iron in the duodenal epithelium has been reported in sex-linked anemia (Sla) mice due to a mutation in the intestinal ferroxidase *Hephaestin* [22]. Based on our data, it is likely that PrP functions upstream from hephaestin, perhaps as a ferric-reductase, to reduce Fe(III) iron stored in ferritin to the Fe(II) form for transport across the BL membrane and subsequent oxidation by hephaestin for delivery to circulating Tf. It is interesting to note that PrP has been reported to perform a similar function in copper transport where it reduces copper (II) prior to its delivery to cytosolic copper (I) carrying proteins [23]. It seems unlikely that PrP is an active ferroxidase based on a previous report [13] and our current observations on the rate of iron efflux from peritoneal macrophages of PrP^KO^ mice. However, differential activity of PrP in different cell lines cannot be ruled out, leaving it unclear whether PrP indeed functions as a ferroxidase like hephaestin. Since the iron deficiency in PrP^KO^ mice is largely compensated and the animals live normally except for delayed erythropoiesis after induced hemolysis and specific deficiencies restricted mainly to the central nervous system [5], [12], it is likely that PrP modulates the function of other iron uptake proteins or is involved in a parallel pathway that compensates for its absence. Identification of such proteins and pathways would be a significant step forward in understanding the role of PrP in iron export from the duodenum.

Absence of PrP also compromises the ability of hematopoietic precursor cells to take up iron from plasma Tf, an interesting observation since these cells acquire most of their iron by the Tf/TfR pathway. A similar phenotype of iron deficiency has been reported in neuroblastoma cells expressing lower levels or mutant forms of PrP, another cell line that utilizes the Tf/TfR pathway for acquiring iron [13]. It is therefore likely that PrP functions at a point where the enterocyte and the Tf/TfR pathway of iron uptake and transport intersect. Normally, plasma Tf provides iron to most cells of the body, and itself receives iron from three main sources; 1) food via the duodenum and proximal jejunum, 2) recycled senescent RBCs, and 3) iron stores within macrophages and hepatocytes. Most of the iron taken up from food is utilized for erythropoiesis, and RBCs contain 80% of the total body iron. Iron rich Tf binds to the TfR on hematopoietic precursor cells, and the Tf/TfR complex is endocytosed through clathrin mediated endocytosis. Tf bound iron is released in the acidic environment of the endosomes, reduced by the ferric reductase Steap3, and transported across the membrane by DMT1 to cytosolic ferritin for storage. Ferritin maintains the cellular labile iron pool to provide for the metabolic needs of cells, and is itself regulated by the iron content of cells through iron management proteins [22]–[26]. It is surprising that the mere absence of PrP compromises the ability of parenchymal cells of various organs and hematopoietic precursor cells to take up iron.

PrP^KO^ mice show an earlier and a significantly higher spike in ^59^Fe uptake by the spleen and long bones relative to Wt controls. Since RBCs consume the majority of plasma iron and the spleen and long bones are the principal sites of erythropoiesis, these findings reflect an abnormally high rate of erythropoiesis in PrP^KO^ mice. The presence of ^59^Fe labeled cells in the peripheral blood of PrP^KO^ mice after four hours of chase is surprising given the time it takes for RBCs to mature, and may reflect the binding of ^59^Fe-Tf to TfR positive immature erythrocytes. However, while Wt RBCs continue to incorporate ^59^Fe, PrP^KO^ RBCs show only a modest increase even after eleven days of chase. These results suggest that the erythropoiesis in PrP^KO^ mice is ineffective despite adequate supplies of iron in the spleen and long bones, probably due to inefficient uptake by the hematopoietic precursor cells. The alternate possibility, i.e., increased turnover of RBCs in the spleen or intravascular hemolysis is unlikely based on the following observations: 1) ^59^Fe counts did not increase in the spleen of PrP^KO^ mice at any time point after four hours of chase, the main organ where RBCs are turned over and the released iron recycled, 2) PrP^KO^ RBCs did not show a decline in ^59^Fe counts during eleven days of chase given that the normal half-life of mouse RBCs is ∼15 days, 3) ^59^Fe from hemolyzed RBCs was not detected in the urine, and 4) markers of hemolysis such as hemoglobinuria were not detected in PrP^KO^ mice (unpublished observations). Furthermore, the kinetics of ^59^Fe uptake from the plasma of Wt and PrP^KO^ mice suggests decreased uptake by target organs rather than increased hemolysis in the latter. The precise biochemical pathway by which PrP facilitates iron uptake by the parenchymal and hematopoietic precursor cells is difficult to define from our data. However, based on our observations from neuroblastoma cells [13], it is likely that PrP facilitates transport across the endosomal membrane to the cytosol by functioning as a ferric reductase. Further studies are required to resolve this question.

The iron deficiency in PrP^KO^ mice is mild since overt signs of anemia were not detected except for a mild reduction in hemoglobin levels, a significant reduction in serum iron, serum ferritin, and transferrin saturation, and an increase in the total iron binding capacity and the number of circulating reticulocytes. This observation is surprising given the obvious decrease in brain iron content, a privileged organ that is affected last by iron deficiency [27]. It is likely that the response of iron management proteins compensates for the iron deficiency in PrP^KO^ mice in all major organs, including the hematopoietic system. Introduction of PrP on the PrP^KO^ background reverses the iron deficient phenotype completely, indicating that PrP is an integral component of the iron homeostatic machinery that feeds into the major pathways of iron uptake and transport.

In conclusion, this report demonstrates a significant role of PrP in maintaining systemic iron homeostasis. Specifically, PrP modulates iron transport from the duodenum to the blood stream and uptake by cells of the hematopoietic system, brain, liver, and spleen. Since major proteins and pathways of iron transport and uptake utilized by enterocytes and hematopoietic cells differ significantly [26], these observations suggest a functional role for PrP at a point downstream from these pathways. Although our data fall short of identifying the specific role of PrP in iron modulation, this report establishes the functional role of PrP in iron metabolism, and provides essential information on the possible sites where PrP influences iron metabolism independently, or by intersecting with known pathways of iron uptake and transport. Since imbalance of iron homeostasis is a common feature of prion disease affected human, hamster, and mouse brains [14], these results suggest a significant contribution of loss of PrP function to prion disease pathogenesis.

## Materials and Methods

### Materials and antibodies

PrP-specific monoclonal antibody 8H4 (against residues 145 to 180) was obtained from Abcam (Cambridge, MA, USA) and from Drs. Man-Sun Sy and Pierluigi Gambetti (Case Western Reserve University). Rabbit anti-human ferritin antibody that cross-reacts with H and L-chains of mouse ferritin was obtained from Sigma (catalog # F5012, lot number 117K4880), ferritin H-chain specific antibody was from Santa Cruz (Catalog # sc-25617, lot # L0104), ferritin L-chain specific antibody was also from Santa Cruz (Catalog # sc-25616, lot # E0905), anti-Tf was from GeneTex (San Antonio, TX), anti-TfR from Zymed Laboratories Inc (Carlsbad, CA), and horseradish peroxidase (HRP)-conjugated secondary antibodies were from GE Healthcare (Little Chalfont, Buckinghamshire, United Kingdom). All other chemicals were purchased from Sigma.

### Transgenic mice

The PrP^KO^ mice in the FVB background (FVB/*Prnp^0/0^*) were originally obtained from George Carlson, (McLaughlin Research Institute). The PrP^+^ transgenic mice expressing wild type murine *PRNP* were created by microinjection of the half-genomic PrP clone [28] into fertilized FVB/*Prnp^0/0^* eggs. The transgenic mice were screened by PCR and maintained via breeding with FVB/*Prnp^0/0^* mice. The genotype of littermate PrP^KO^ and PrP^+^ mice were determined by PCR of DNA from ear punches.

### Intestinal iron uptake and transport

All procedures with mice were performed according to the guidelines established by the Animal Resource Center of Case Western Reserve University, and were based on protocols approved by the IACUC committee. Three to six month old age- and sex-matched Wt and PrP^KO^ mice maintained under similar conditions were fasted overnight with water *ad libitum*, and 20 µCi of ^59^FeCl_3_ diluted in 0.2 ml of PBS was administered orally with an olive-tipped gavage needle. The mice were chased on normal chow for 1 h, 4 h, 24 h, 48 h and 11 days. At each chase time point, a set of Wt and PrP^KO^ mice were euthanized, and blood was collected by cardiac puncture in heparinized vials. Plasma and red cells were separated by centrifugation at 5,000 rpm for 5 min. Stomach and upper gastro-intestinal tract was separated and rinsed with cold PBS until clean. The samples were placed on a filter paper, vacuum dried, and subjected to autoradiography. The brain, liver, spleen, and femur were collected, washed with PBS, and snap frozen on dry ice. The organs were weighed, and radioactivity was counted in a γ-counter. Selected samples of brain were fixed in 5% phosphate buffered formalin for 24 h, and 700 µM thick sections were cut using a Leica vibrotome. Cut sections were briefly air-dried and exposed to X-ray film.

### Native gradient gel electrophoresis, autoradiography and immunoblotting

Native gradient gel electrophoresis, autoradiography, and immunoblotting were performed essentially as described by Vyoral et al. [29] and in a previous report [13].

### SDS-PAGE and Western blotting

Brain, liver, and spleen homogenates (10%) prepared in lysis buffer were resolved by SDS-PGE and subjected to western blotting using specific antibodies as described in previous reports [30]. Immunoreactive bands were visualized by ECL detection system (Amersham Biosciences Inc.).

### Prussian blue staining

Snap frozen liver and spleen sections (7 µm) were immersed in acidified potassium ferrocyanide solution (4%) for 20 min followed by washing with distilled water. Sections were then counterstained with 1% Neutral Red for 2 min and mounted.

### Determination of plasma and tissue iron

In-gel iron estimation of protein bands and the iron content of liver and spleen homogenates were performed by Ferene-S staining essentially as described [14]. Serum iron, total iron binding capacity and transferrin saturation were determined by using an automated Ferene based detection method on a Dimension RXL chemistry analyzer (Dade Behring, Deerfield, Ill). The CBC and reticulocyte data was determined on a Sysmex XE-2100 hematology analyzer (Kobe, Japan). To detect hemolysis, urine dip-stick was performed on an Urisys 2400 urine analyzer (Roche Diagnostics, Indianapolis IN).

### Transferrin binding assay

Tf binding sites in the brains of Wt and PrP^KO^ mice were assessed by incubating 10 µM thick brain sections prepared from frozen samples essentially as described by Moos et al. [17]. In short, brain sections were pre-incubated with 0.2% bovine serum albumin in PBS for 30 min at 37°C in a humid atmosphere to wash off endogenous Tf, and incubated with FITC-Tf (5 µg/ml) for 30 min at 37°C in a humid chamber. Another set of samples was pre-incubated with apo-transferrin (75 ng) for 10 min at room temperature followed by incubation with FITC-Tf as above. The sections were rinsed with PBS, mounted, and observed using a laser scanning confocal microscope (Bio-Rad MRC 600).

### Estimation of iron export from peritoneal macrophages

Peritoneal macrophages were isolated from Wt and PrP^KO^ mice as described by Hanazawa et al. [18]. Following an overnight culture, adhered macrophages were stimulated with LPS (100 ng/ml) for 24 h, serum starved for 1 h, and labeled with ^59^FeCl_3_-citrate complex (1 mM sodium citrate and 2 µCi of ^59^FeCl_3_ in serum free DMEM for 4 h at 37°C. The molar ratio of citrate to iron was maintained at 100∶1. Cell surface bound iron was washed and the cells were chased in complete medium for different time periods. A 100 µl aliquot of the medium was retrieved at each time point and counted in a γ-counter. After 18 h the cells were lysed and cell associated ^59^Fe was measured in a γ-counter.

### Statistical analysis

A minimum of 3 mice in each group were analyzed for all experiments, and the experiments were repeated at least 3 times. The results are expressed as mean±standard error of mean (SEM). Statistical analysis was done by unpaired Student's t test when comparing Wt with PrP^KO^ group. For comparing Wt, PrP^KO^ and littermates groups, one way ANOVA followed by Bonferroni multiple comparison post hoc test was done using GraphPad Prism software (Version 5.02, GraphPad Inc., San Diego, CA, USA). Differences were considered significant at *p*<0.05.
